# Cryogel-Immobilized Catalase as a Biocatalyst with Enhanced Stability Against Microplastics

**DOI:** 10.3390/gels11080634

**Published:** 2025-08-12

**Authors:** Kadir Erol, Mehmet Hüseyin Alkan, İhsan Alacabey

**Affiliations:** 1 Department of Medical Services and Techniques, Vocational School of Health Services, Hitit University, 19030 Çorum, Turkey; kadirerol86@gmail.com; 2 Department of Basic Pharmaceutical Sciences, Faculty of Pharmacy, Dicle University, 21280 Diyarbakır, Turkey; 3 Department of Medical Services and Techniques, Vocational School of Health Services, Mardin Artuklu University, 47100 Mardin, Turkey; ihsanalacabey@hotmail.com

**Keywords:** catalase immobilization, cryogel matrix, microplastic stress

## Abstract

Catalase is a pivotal antioxidant enzyme that decomposes hydrogen peroxide and reduces oxidative stress. However, its low thermal and operational stability limits applications in challenging environments, particularly those contaminated with emerging pollutants such as polystyrene-based microplastics (PS-MPs). In this study, cryogels composed of Poly(2-hydroxyethyl methacrylate-co-allyl glycidyl ether) [Poly(HEMA-co-AGE)] were synthesized and evaluated as immobilization matrices to enhance catalase stability. Cryogels containing varying AGE concentrations were characterized using FT-IR, SEM, TEM, TGA, and BET analyses. The formulation with 250 µL AGE exhibited optimal physicochemical properties, including improved water retention, increased surface area, and high immobilization capacity (356.3 mg·g^−1^). Immobilized catalase maintained superior activity under PS-MP-induced stress across a range of concentrations (0–1.0 mg·mL^−1^), temperatures (4–60 °C), and exposure times (up to 5 h). Kinetic modeling revealed a significant improvement in substrate affinity, with Km decreasing from 54.9 to 17.1 mM, while Vmax decreased moderately. Long-term stability tests showed that immobilized catalase retained ~80% activity after 70 days at 4 °C and 55% after 15 reuse cycles. Desorption studies confirmed the reusability of the cryogel system. These findings suggest that Poly(HEMA-co-AGE) cryogels provide a robust and reusable platform for catalase stabilization, offering potential for applications such as wastewater treatment and biosensing in microplastic-contaminated systems.

## 1. Introduction

Catalase is a key antioxidant enzyme in various aerobic organisms, including bacteria, fungi, plants, and mammals [1]. In mammalian cells, it is primarily localized in peroxisomes, decomposing hydrogen peroxide (H_2_O_2_) into water and oxygen, thereby mitigating oxidative stress [2]. Although catalase enzymes across species share a standard tetrameric, heme-containing structure, variations in molecular organization, cellular localization, and functional properties lead to differences in substrate specificity and catalytic performance [3]. Some catalases are monofunctional, while others possess bifunctional or manganese-dependent activity, further diversifying their biological roles [4].

Although catalase plays a vital physiological role, its industrial applications are hindered by low thermal and pH stability, limited activity in aqueous systems, and rapid inactivation after a single use. Enzyme immobilization has emerged as a promising approach to overcome these limitations [5]. By anchoring the enzyme onto solid supports, immobilization enhances enzyme stability, reusability, and resistance to denaturation. Various immobilization techniques (such as adsorption, entrapment, and covalent bonding) have been explored using materials such as metal oxides, nanofibers, and magnetic nanoparticles to retain or improve catalytic activity [6,7,8,9]. However, immobilization may also induce conformational changes that hinder substrate access and alter kinetic parameters, often increasing the Michaelis–Menten constant (*K_m_*) and reducing the maximum reaction rate (*V_max_*) [10].

Recent developments have introduced advanced immobilization strategies, including using intermediary agents and 3D-printed platforms to create tailored support structures. These innovations aim to improve immobilization efficiency while preserving the enzyme’s native conformation and functionality. Immobilized catalase has demonstrated potential in various fields, such as wastewater treatment, pharmaceuticals, food processing, and diagnostics [11]. Nonetheless, its large molecular size, tetrameric structure, and deeply embedded active sites continue to pose challenges by limiting efficient electron transfer and catalytic activity. As a result, ongoing research focuses on novel immobilization techniques to fully exploit catalase’s biocatalytic potential [12].

Cryogels, macroporous polymers formed by cryopolymerization at sub-zero temperatures, offer an up-and-coming platform for enzyme immobilization. Their interconnected porous structures facilitate high mass transfer, mechanical robustness, and rapid solute diffusion, which are advantageous in both adsorption and biocatalysis [13,14,15]. Due to their tunable surface properties and inherent hydrophilicity, cryogels exhibit high affinity for diverse pollutants, including dyes, heavy metals, and pharmaceuticals [16]. Their stable physical architecture provides an optimal enzyme microenvironment, preserving catalytic activity while enhancing thermal and operational stability [17]. Furthermore, their reusability and ease of regeneration support their role in sustainable biotechnological applications [18]. Recent studies have demonstrated the efficacy of functional cryogels in environmental remediation and enzyme stabilization, particularly for biocatalysts such as catalase and laccase [19,20]. Developing nanocomposite- and amino acid-based cryogels has advanced this field by improving biocompatibility, selectivity, and fouling resistance [21,22]. As multifunctional materials, cryogels bridge high-efficiency separation systems and green, reusable bioprocessing platforms [23].

Polystyrene microplastics (PS-MPs), prevalent in aquatic and terrestrial ecosystems, pose significant threats to biological systems due to their potential to interact with proteins and enzymes [24,25]. These interactions can lead to conformational disruptions, surface adsorption, and obstruction of active sites, ultimately impairing enzymatic activity [26]. Experimental studies have shown that PS-MPs induce oxidative stress and reduce the catalytic efficiency of key antioxidant enzymes, such as catalase and superoxide dismutase [27,28]. Such disruptions weaken cellular antioxidant defenses and highlight the urgent need to study enzyme–microplastic interactions and their ecological consequences [29,30].

In this study, catalase was selected as a model enzyme due to its well-established antioxidant activity and relevance in environmental applications, particularly wastewater treatment. Despite its potential, catalase often suffers from low stability under harsh conditions, limiting its practical use. To address this, Poly(2-hydroxyethyl methacrylate-co-allyl glycidyl ether) [Poly(HEMA-co-AGE)] cryogels were synthesized and employed as immobilization matrices. This polymer system was chosen for its high water retention, interconnected macroporous structure, and reactive epoxy groups, all of which create a favorable microenvironment for enzyme stabilization and improved performance. The activity of free and immobilized catalase was evaluated under various stress conditions, including different concentrations of polystyrene-based microplastics, incubation times, and temperatures. Whereas microplastic exposure severely diminished the activity of free catalase, the immobilized enzyme exhibited improved resistance to inhibition. These findings highlight the potential of Poly(HEMA-co-AGE) cryogels as robust and reusable platforms for enzyme immobilization and as practical tools for mitigating the impact of emerging environmental pollutants, such as microplastics.

## 2. Results and Discussions

### 2.1. Characterizations

**Figure 1:**
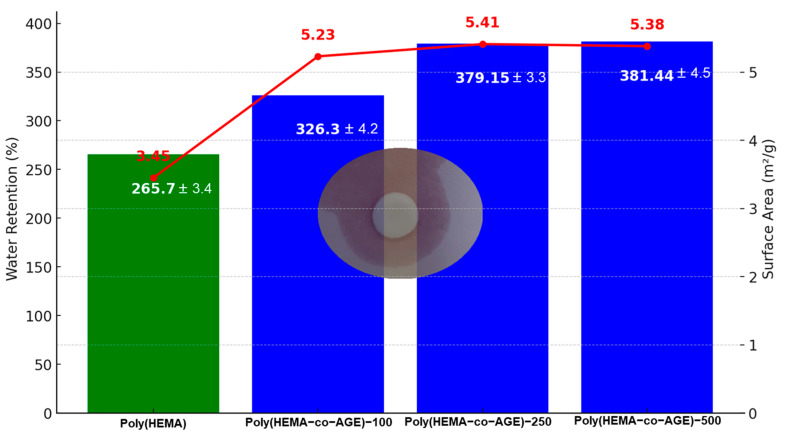
Water retention capacities and surface areas of Poly(HEMA) and Poly(HEMA-co-AGE) cryogels synthesized with different AGE contents (100, 250, and 500 µL). Data are presented as the mean ± standard deviation from three independent experiments (*n* = 3).

The graph presents a comparative evaluation of the water retention capacity (%) and specific surface area (m^2^/g) of Poly(HEMA) and Poly(HEMA-co-AGE) cryogels synthesized with varying AGE contents (100, 250, and 500 µL) (Figure 1). The incorporation of AGE into the Poly(HEMA) matrix significantly enhances both parameters. Poly(HEMA) cryogels showed the lowest water retention (265.7%) and surface area (3.45 m^2^/g), suggesting a compact structure with low porosity and hydrophilicity.

In contrast, all AGE-modified cryogels showed notable improvements. Among them, Poly(HEMA-co-AGE)-250 demonstrated the highest surface area (5.41 m^2^/g), while Poly(HEMA-co-AGE)-500 exhibited the most significant water retention capacity (381.44%). The simultaneous enhancement in both parameters suggests that AGE incorporation increases porosity and hydrophilic interactions, promoting improved swelling behavior and increased accessible surface areas. Interestingly, although the surface area plateaued between AGE-250 and AGE-500, water retention continued to rise slightly, implying that additional AGE may contribute more to water-binding efficiency than further surface area increases [31].

Overall, the data strongly support that AGE incorporation enhances the physical structure and functional properties of the cryogels, rendering Poly(HEMA-co-AGE) systems more suitable for applications requiring high water absorption and surface-mediated interactions, such as biocatalysis, drug delivery, adsorption-based separations, and enzyme immobilization.

**Figure 2:**
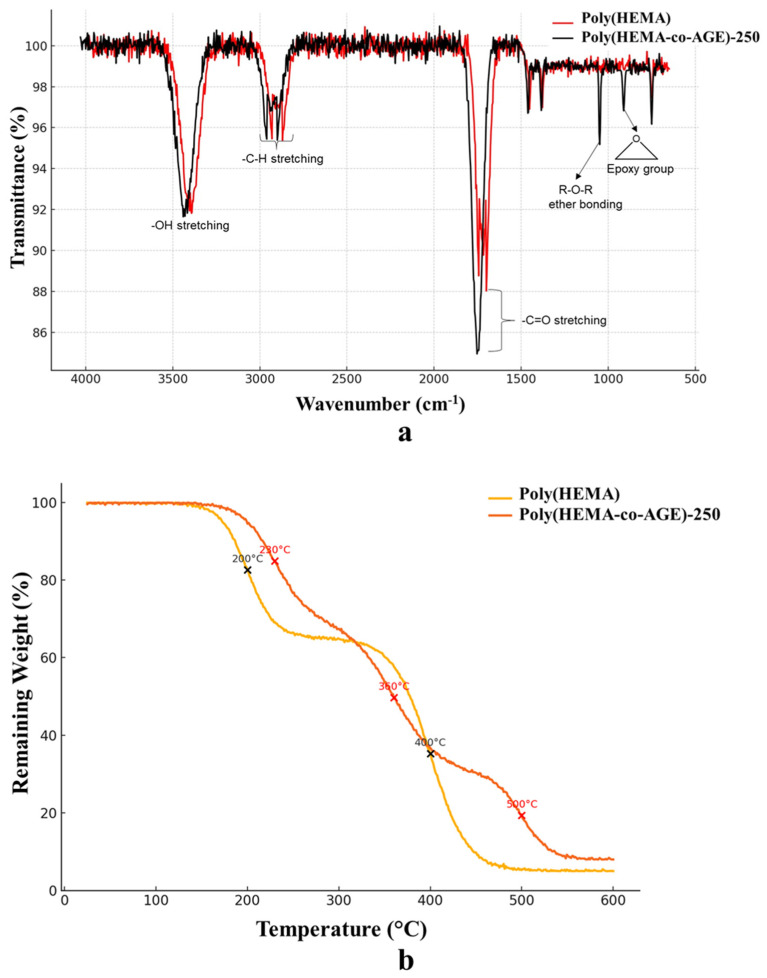
(**a**) FT-IR spectra and (**b**) TGA curves of Poly(HEMA) and Poly(HEMA-co-AGE)-250 cryogels, confirming successful AGE incorporation and differences in thermal stability. Spectra and thermograms are representative of three independently synthesized samples (*n* = 3), demonstrating reproducibility across batches.

The comparative FT-IR spectra of Poly(HEMA) and Poly(HEMA-co-AGE) cryogels (Figure 2a) confirm the successful incorporation of AGE into the polymer network. Both spectra exhibit characteristic bands corresponding to –OH stretching (~3400 cm^−1^), C–H stretching (~2900 cm^−1^), and C=O stretching (~1720 cm^−1^), which are typical of HEMA-based polymers. However, the Poly(HEMA-co-AGE) spectrum features additional peaks around 910 cm^−1^ and 840 cm^−1^, attributed to the epoxy functional groups introduced by AGE. A more pronounced band near 1100 cm^−1^, indicative of R–O–R ether linkages, further supports the presence of glycidyl units. These spectral features, either absent or significantly weaker in the neat Poly(HEMA), are clear evidence of successful copolymerization and the introduction of reactive moieties for further crosslinking or functionalization [32].

The TGA of Poly(HEMA) and Poly(HEMA-co-AGE) cryogels (Figure 2b) reveals distinct multi-step degradation patterns reflecting differences in chemical structure and crosslinking density. The Poly(HEMA) cryogel undergoes two significant weight losses around 200 °C and 400 °C, corresponding to the loss of physically entrapped moisture and the degradation of the polymer backbone, respectively [33]. The final residue (~5%) suggests relatively low thermal stability.

Conversely, Poly(HEMA-co-AGE) cryogels display a three-step degradation profile with essential weight losses centered at approximately 230 °C, 360 °C, and 500 °C. The inclusion of AGE likely increases the structural complexity and introduces additional thermally labile segments and crosslinks, delaying the onset of degradation and broadening the decomposition range. A higher residual weight (~8%) further indicates improved char formation and enhanced thermal resistance. These changes suggest that AGE-modified cryogels are more thermally robust, which is advantageous for applications involving elevated temperatures or prolonged operational stability.

**Figure 3:**
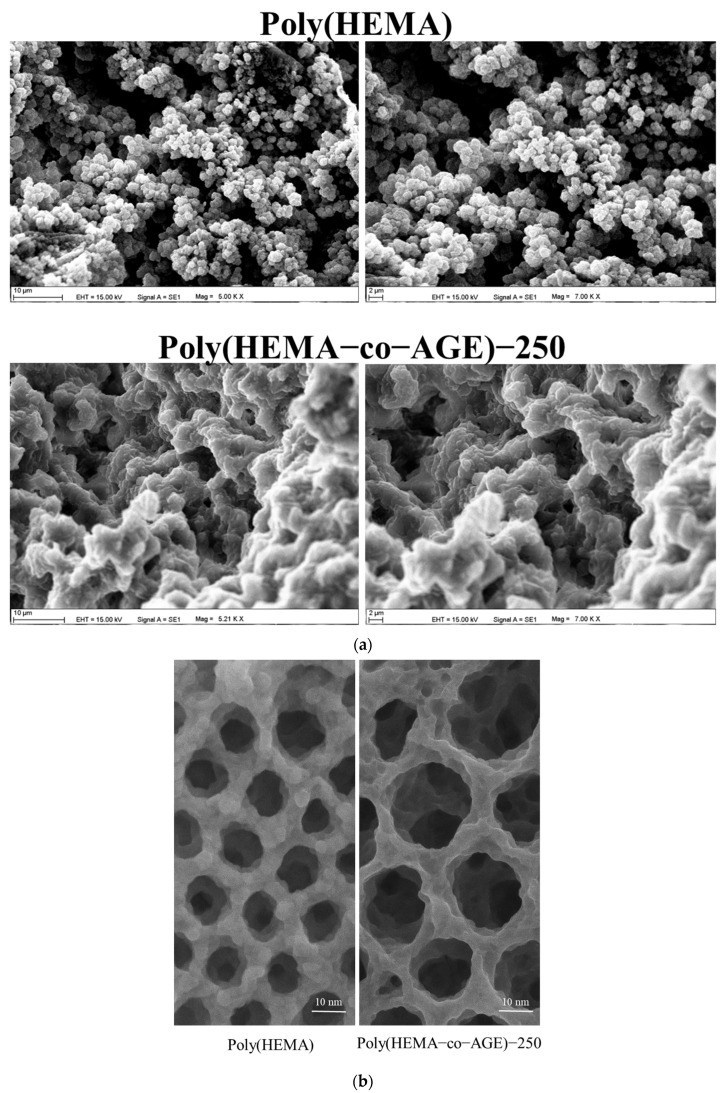
(**a**) SEM and (**b**) TEM images of Poly(HEMA) and Poly(HEMA-co-AGE)-250 cryogels. All images are representative of at least three independently synthesized cryogel samples (*n* = 3). TEM measurements revealed average pore diameters of approximately 8–12 nm for Poly(HEMA) and 15–25 nm for Poly(HEMA-co-AGE)-250, consistent with structural differences observed in replicate preparations.

SEM images (Figure 3a) illustrate distinct morphological differences between the cryogels; however, they are not intended to reveal the localization or distribution of immobilized catalase or PS-MPs within the matrix. Poly(HEMA) exhibits a compact and uniform porous structure with small, well-defined pores, indicating high mechanical stability but limited surface accessibility. In contrast, the Poly(HEMA-co-AGE) cryogel exhibits a more open, interconnected, and irregular pore network, which promotes improved mass transport and surface interactions, critical for effective adsorption and enzyme immobilization. The incorporation of AGE introduces functional epoxy groups, providing reactive sites for molecular binding [32]. Despite appearing more porous in SEM images, Poly(HEMA) actually exhibits a lower surface area due to its dense, less interconnected structure. As shown in Figure 2, Poly(HEMA-co-AGE) cryogels exhibit a significantly higher specific surface area. This discrepancy is attributed to the 3D interconnected morphology of Poly(HEMA-co-AGE), which is less evident in 2D SEM projections but contributes substantially to surface availability, as confirmed by BET analysis.

TEM images (Figure 3b) further support these observations. Poly(HEMA) cryogels feature smaller and more uniform pores, approximately 8–12 nm in diameter, reflecting a denser internal architecture. In comparison, Poly(HEMA-co-AGE)-250 cryogels show significantly larger (15–25 nm), irregular, and sponge-like interconnected pores, suggesting increased porosity and superior diffusion characteristics. These structural improvements support the suitability of AGE-modified cryogels for environmental cleanup, biosensing, and biomedical use.

### 2.2. Effect of Immobilization Time and AGE Content on the Catalase Loading Efficiency of Poly(HEMA-co-AGE) Cryogels

**Figure 4:**
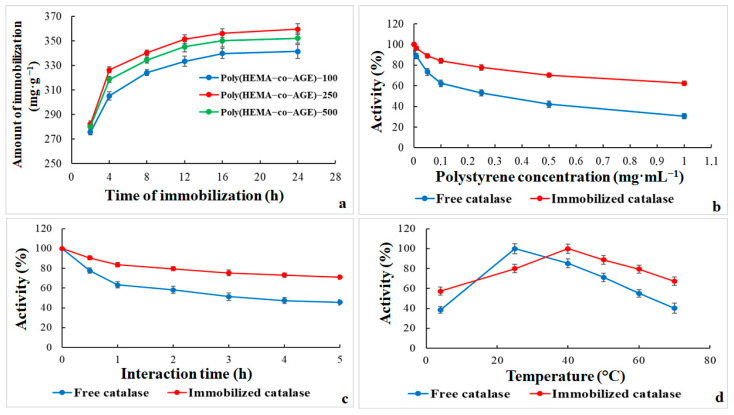
(**a**) Effect of immobilization time on the catalase loading capacity of Poly(HEMA-co-AGE) cryogels synthesized with different AGE contents (100, 250, and 500 µL). (**b**) Relative enzymatic activity of free and immobilized catalase after 1 h of interaction with varying concentrations (0–1.0 mg·mL^−1^) of PS-MPs at room temperature. (**c**) Time-dependent enzymatic activity changes over a 5 h interaction period with a 0.1 mg·mL^−1^ polystyrene microparticle suspension at room temperature. (**d**) Temperature-dependent catalase activity in the presence of 0.1 mg·mL^−1^ PS-MPs following 1 h of interaction. The Poly(HEMA-co-AGE)-250 cryogel was used in the immobilization studies in graphs (**b**–**d**). All data represent the mean values of at least three independent experiments.

The graph in Figure 4a illustrates the effect of immobilization time on the amount of catalase immobilized (mg·g^−1^) onto Poly(HEMA-co-AGE) cryogels containing varying amounts of AGE (100, 250, and 500 µL). For all three cryogel formulations, catalase loading rose rapidly within the first 4–8 h, likely due to abundant active sites and efficient enzyme diffusion into the porous matrix. Among the tested formulations, Poly(HEMA-co-AGE)-250 showed the highest enzyme loading across all time points, reaching ~360 mg·g^−1^ at 24 h. Poly(HEMA-co-AGE)-500 demonstrates slightly lower, yet still substantial, immobilization efficiency, whereas Poly(HEMA-co-AGE)-100 shows the lowest performance, plateauing at around 340 mg·g^−1^.

These proofs suggest that the AGE content significantly affects enzyme binding efficiency, with 250 µL emerging as the optimal concentration. Increasing the AGE content to 500 µL may lead to steric hindrance or a lower porosity, reducing enzyme access and diffusion [34]. Furthermore, the immobilization capacity levels off after 12 h, indicating the saturation of available binding sites or the attainment of equilibrium in the enzyme-loading process. Poly(HEMA-co-AGE)-250 generally appears to be the most suitable matrix for catalase immobilization, with 16 h being optimal.

### 2.3. The Kinetic Parameters of Free and Immobilized Catalase

The kinetic parameters, including the *K_m_* and *V_max_*, for both free and immobilized catalase on Poly(HEMA-co-AGE)-250 cryogels were determined using Lineweaver–Burk plots, with hydrogen peroxide (H_2_O_2_) as the substrate. *V_max_* reflects the maximum enzymatic activity when the enzyme is fully saturated with the substrate, providing insight into the whole catalytic efficiency. However, it can be affected by mass transfer limitations. *K_m_* represents the substrate concentration at which the reaction rate reaches half of *V_max_* and indicates the enzyme’s substrate affinity.

This study calculated the *K_m_* value for free catalase as 54.9 mM. In comparison, immobilized catalase exhibited a substantially lower *K_m_* of 17.1 mM, indicating an approximately 3.2-fold increase in substrate affinity upon immobilization. In contrast, the *V_max_* of free catalase was 2433 µmol·min^−1^, whereas that of the immobilized form decreased to 1108 µmol·min^−1^ (Table 1), demonstrating that immobilization markedly influenced the catalytic behavior of the enzyme.

The lower *K_m_* indicates improved substrate affinity, likely due to conformational stabilization of the enzyme in the cryogel matrix. However, the lower *V_max_* may result from diffusional limitations, steric hindrance, or partial conformational constraints arising from interactions between the enzyme and the polymer network [34]. These factors may restrict substrate access or cause minor structural changes, leading to reduced catalytic efficiency.

### 2.4. Protective Effects of Cryogel Immobilization on Catalase Activity Against Polystyrene Microplastics and Thermal Stress

Figure 4b–d demonstrates the inhibitory effects of increasing concentrations of PS-MPs (0–1.0 mg·mL^−1^) on the relative enzymatic activity of both free and immobilized catalase. A concentration-dependent decline in activity is observed for both enzyme forms, indicating that PS-MPs adversely affect catalase function (Figure 4b). However, the extent of inhibition significantly differs between the two forms.

At 0.1 mg·mL^−1^ polystyrene, the activity of free catalase decreases sharply to approximately 62.5%, while immobilized catalase retains over 84% of its initial activity. At 1.0 mg·mL^−1^ PS-MPs, free catalase activity falls to 30.6%, while the immobilized form retains 62.5%, highlighting the cryogel’s protective role. These findings indicate the shielding role of the cryogel matrix, which likely limits the diffusion of microparticles and reduces direct interaction with the enzyme’s active site [19]. In contrast, free catalase is more vulnerable to adverse effects such as surface adsorption, conformational destabilization, and oxidative interactions induced by polystyrene.

From an application standpoint, this enhanced resistance of immobilized catalase to micropollutant-induced deactivation supports cryogel-based immobilization platforms in contaminated environments, particularly wastewater systems containing microplastics. The results emphasize the critical role of immobilization in preserving enzymatic activity under environmental stressors [32,34,35].

Figure 4c further illustrates the time-dependent impact of PS-MPs on the catalytic activity of free and immobilized catalase over 5 h of exposure. Initially (0 h), both enzyme forms display full relative activity (100%), indicating no immediate interaction. Over time, however, a notable divergence in stability emerges. Free catalase rapidly declines, decreasing to about 63.3% within the first hour and 45.5% by the fifth hour. This pronounced inactivation is likely due to direct, unmediated interactions with polystyrene, leading to structural distortion, nonspecific adsorption, or localized oxidative stress.

In comparison, immobilized catalase demonstrates superior stability, retaining approximately 83.5% activity after 1 h and 70.9% after 5 h. The cryogel likely forms a protective microenvironment that limits particle access to the active site, stabilizes the enzyme’s structure, and reduces nonspecific interactions. These results underscore the added value of immobilization in sustaining enzymatic performance under prolonged microparticle exposure [34,35].

Figure 4d presents the temperature-dependent activity profiles of free and immobilized catalase in the presence of PS-MPs. Free catalase activity increases rapidly and peaks as the temperature rises from 4 °C to 25 °C. In contrast, immobilized catalase gradually increases, achieving maximum activity (100%) at 40 °C. This pattern indicates that immobilization improves thermal resistance, allowing catalase to function efficiently at elevated temperatures.

Beyond their respective optimal temperatures, both enzyme forms show decreasing activity. Nonetheless, immobilized catalase consistently retains a higher percentage of activity above 30 °C. For example, at 60 °C, free catalase activity falls to approximately 55.1%, while the immobilized enzyme retains around 79.4%. This improved thermal resilience is likely due to the structural rigidity imposed by the cryogel matrix, which reduces conformational flexibility and protects against thermally induced unfolding and microparticle interference [32].

In conclusion, the data collectively demonstrate that enzyme immobilization substantially mitigates the adverse effects of both thermal and micropollutant stress. Immobilized catalase shows enhanced stability, making it a more robust candidate for biocatalytic applications under variable and contaminated environmental conditions.

The observed protective effect of the Poly(HEMA-co-AGE)-250 cryogel matrix against PS-MP-induced enzymatic inhibition is hypothesized to arise from both physical and biochemical factors. The interconnected macroporous structure likely acts as a physical barrier, restricting the diffusion and direct access of PS-MPs to the immobilized enzyme. Furthermore, the covalent immobilization of catalase via reactive epoxy groups may stabilize the enzyme’s conformation, reducing its susceptibility to denaturation or surface adsorption induced by microparticles. These combined effects help preserve the active site’s structural integrity and catalytic function under stress conditions. Although the current findings are supported by morphological (SEM/TEM), kinetic, and activity data, further investigations using spectroscopic or molecular modeling techniques are warranted to provide deeper insight into the molecular mechanisms underlying this protective behavior.

### 2.5. Stability and Reusability Studies

**Figure 5:**
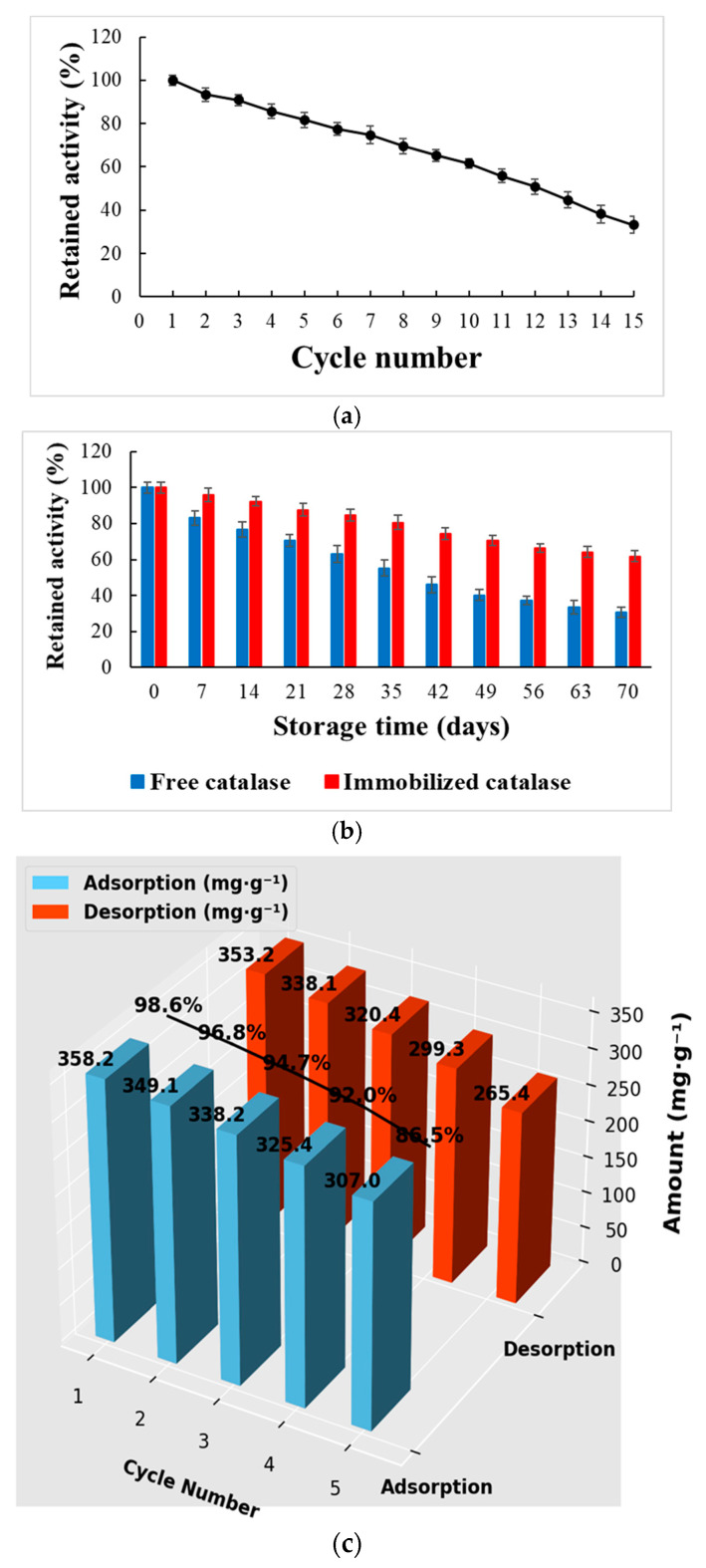
Stability and reusability of immobilized catalase on Poly(HEMA-co-AGE)-250 cryogels: (**a**) retained activity over 15 reuse cycles; (**b**) storage stability at 4 °C over 70 days for free and immobilized catalase; and (**c**) adsorption and desorption capacities over five cycles, indicating high reusability and recovery efficiency. All data represent the mean values of at least three independent experiments.

The catalytic performance and stability of catalase immobilized onto Poly(HEMA-co-AGE)-250 cryogels were thoroughly evaluated through operational stability, storage stability, and reusability assessments. Operational testing revealed that immobilized catalase retained ~55% of its initial activity after 15 successive cycles at 25 °C (Figure 5a). The cryogel disks were regenerated between each cycle by using phosphate buffer to remove residual substrates and products. The gradual decline in activity is likely attributable to enzyme leaching or partial conformational changes; nevertheless, maintaining activity over 50% demonstrates strong structural support and protective interactions provided by the cryogel matrix. These results confirm the ability of the cryogel network to preserve enzymatic activity under extended operational conditions [34,35], which is critical for industrial biocatalytic processes.

Storage stability was assessed by comparing free and immobilized catalase over 70 days at 4 °C (Figure 5b). While the free enzyme exhibited a significant decrease in activity, retaining only about 40% of its initial value, the immobilized form maintained nearly 80% of its original activity. This substantial difference underscores the stabilizing effect of the cryogel matrix, which likely protects the enzyme from environmental degradation, conformational instability, and denaturation during storage [32,34,35]. The extended shelf life of the immobilized enzyme is particularly beneficial for applications that demand long-term storage and repeated use.

Additionally, the reusability and desorption efficiency of the cryogel system were examined to assess its practical applicability (Figure 5c). Adsorption–desorption studies revealed high enzyme loading capacities, ranging from 358.2 to 307.0 mg·g^−1^, and desorption efficiencies between 98.6% and 86.5% over five cycles using 1 M NaCl as the eluent [34]. Although FT-IR data alone cannot confirm the exact binding nature, the high retention of enzyme activity after multiple cycles and low desorption in 1 M NaCl suggest that catalase is primarily immobilized via covalent interactions with the epoxy groups of the cryogel matrix. While the observed decrease in *V_max_* upon immobilization (Section 2.3) is primarily attributed to mass transfer limitations within the cryogel matrix, additional factors may also contribute. The possibility of partial enzyme inactivation during the immobilization process or limited accessibility of the active sites due to covalent binding cannot be entirely excluded. However, the immobilization procedure was carried out under mild conditions (4 °C, pH 7.0), and no sudden activity loss was detected during enzyme loading or reusability assays. Moreover, desorption studies indicated a high enzyme recovery rate (≥86.5%) after multiple cycles, suggesting minimal enzyme leaching and preserved structural integrity. Nevertheless, slight conformational constraints or steric hindrance arising from the polymeric microenvironment may partially reduce catalytic turnover, which would help explain the significant drop in *V_max_* despite improved substrate affinity. These results indicate that the Poly(HEMA-co-AGE)-250 cryogel facilitates efficient enzyme immobilization and recovery with minimal structural deterioration, enabling repeated use in immobilization–desorption processes.

In summary, Poly(HEMA-co-AGE)-250 cryogels represent a robust and efficient platform for catalase immobilization, offering enhanced stability under both operational and storage conditions. Their high reusability and excellent enzyme recovery performance underscore their suitability for sustainable and cost-effective applications in industrial biotechnology and environmental biocatalysis. Notably, the high enzyme recovery efficiency (≥86.5%) observed after desorption suggests that the cryogel system can be effectively regenerated and reloaded with enzyme, thereby extending its usability for repeated or continuous use in long-term processes.

### 2.6. Comparative Evaluation of Catalase Immobilization on Various Cryogel Systems

Table 2 presents a comparative analysis of various cryogel matrices employed for catalase immobilization, evaluating key parameters such as immobilization capacity, kinetic constants (*K_m_* and *V_max_*), operational and storage stabilities, and reusability.

Among the materials examined, the Poly(HEMA-co-AGE)-250 cryogel synthesized in the present study exhibits the highest immobilization capacity (356.3 ± 3.6 mg·g^−1^), reflecting a superior enzyme loading efficiency. This is followed by Poly(HEMA-GMA) [34] and p(HEMA)-BPCGO [35], with capacities of 298.7 ± 9.9 mg·g^−1^ and 261.7 ± 11.2 mg·g^−1^, respectively. In contrast, Fe^3+^-poly(AAm-GMA)-IDA [36] shows the lowest immobilization capacity (12.99 mg·g^−1^), likely due to limited functional groups or suboptimal surface interactions.

Kinetic analyses further reveal the impact of immobilization on enzyme–substrate interactions. A notable decrease in *K_m_*, as seen in Poly(HEMA-GMA) (from 10.52 to 5.43 mM) [34], indicates improved substrate affinity. Conversely, an increase in *K_m_*, such as in p(HEMA)-BPCGO (from 9.9 to 11 mM) [35], may reflect hindered substrate diffusion within the cryogel structure. The Poly(HEMA-co-AGE)-250 cryogel displays a pronounced decrease in *K_m_* (from 54.9 to 17.1 mM), suggesting a substantial enhancement in substrate binding following immobilization.

Changes in *V_max_*, representing maximum catalytic activity, vary across cryogels. For instance, p(HEMA)-BPCGO shows an increase (from 357.1 to 769.2 µmol·min^−1^), possibly due to the conformational stabilization of the immobilized enzyme. In contrast, Poly(HEMA-co-AGE) exhibits a reduction in *V_max_* (from 330 to 8.7 µmol·min^−1^), which may result from structural constraints or limited substrate diffusion [32].

Concerning operational stability, Poly(HEMA-co-AGE)-250 retains 33.1% of its initial activity after 15 cycles, closely matching p(HEMA)-BPCGO, which retains 34.4%. Fe^3+^-poly(AAm-GMA)-IDA notably demonstrates exceptional reusability, preserving 96.7% of its activity after 40 cycles [36].

Storage stability is a crucial parameter for industrial implementation. The Poly(HEMA-co-AGE)-250 cryogel maintains 80.6% of its initial activity after 35 days at 4 °C, outperforming many previously reported systems, such as p(HEMA)-BPCGO, which retains 65.12% over 15 days [35]. This suggests enhanced enzyme stabilization within the matrix. Furthermore, early-cycle reusability remains high across all cryogels, with Poly(HEMA-co-AGE)-250 retaining 85.69% of its activity after five reuse cycles, underscoring its practical applicability.

While AGE-based cryogels were used in previous studies [32], this work is the first to systematically identify 250 µL of AGE as the optimal monomer concentration for enhanced catalase immobilization. In particular, the observed protective effect of the cryogel matrix against polystyrene-induced enzymatic inhibition adds a novel dimension to its practical utility. Among the evaluated cryogels, the Poly(HEMA-co-AGE)-250 formulation stands out for its superior enzyme loading capacity and excellent long-term stability. Collectively, these features underscore its strong potential for catalase-based applications in industrial biocatalysis and environmental remediation.

## 3. Conclusions

This study introduces Poly(HEMA-co-AGE) cryogels as efficient platforms for catalase immobilization, effectively addressing critical limitations in enzyme stability and reusability under environmentally challenging conditions, particularly in the presence of polystyrene microplastics. Incorporating AGE into the cryogel structure was found to enhance water retention, surface areas, and enzyme-binding capacity, with the 250 µL AGE formulation exhibiting the most favorable performance.

Catalase immobilized within the cryogels displayed significantly greater resistance to activity loss under microplastic exposure, maintaining up to twice the relative activity of free catalase across various concentrations and exposure durations. Kinetic analysis revealed a marked decrease in *K_m_*, indicating improved substrate affinity post-immobilization. The observed reduction in *V_max_* was attributed to diffusional limitations and structural constraints imposed by the cryogel matrix. Moreover, the immobilized enzyme retained approximately 80% of its initial activity after 70 days of storage and sustained robust catalytic performance over 15 reuse cycles. High desorption efficiency with NaCl solution further confirmed the potential for enzyme recovery and reuse.

The observations highlight the protective role of cryogel immobilization against polystyrene-induced enzymatic inhibition and thermal degradation. The dual functionality of Poly(HEMA-co-AGE) cryogels, providing both stability and reusability, makes them excellent candidates for real-world applications, such as wastewater treatment and biosensor development. Compared to previously reported cryogel systems, the Poly(HEMA-co-AGE)-250 formulation offers exceptional immobilization capacity along with well-balanced kinetic and stability features. Collectively, this work underscores the potential of functional cryogel matrices to advance sustainable, high-performance biocatalytic technologies for environmental remediation.

## 4. Materials and Methods

### 4.1. Chemicals

Microparticles based on polystyrene (10 μm), catalase from bovine liver, 2-hydroxyethyl methacrylate (HEMA, 98%), allyl glycidyl ether (AGE, ≥99%), N,N′-methylenebisacrylamide (MBAAm, 99%), N,N,N′,N′-tetramethylethylenediamine (TEMED, ≥99.5%), ammonium persulfate (APS, ≥99.99%), potassium dihydrogen phosphate, potassium hydrogen phosphate, hydrogen peroxide solution (30%), and sodium chloride (NaCl, ACS reagent, ≥99.0%) were all purchased from Sigma-Aldrich (Steinheim, Germany). Unless otherwise stated, all other chemicals used in this study were of analytical grade. Ultra-pure water with a resistivity of 18.2 MΩ·cm was used throughout all experimental procedures.

### 4.2. Synthesis of Poly(HEMA-co-AGE) Cryogels

**Figure 6:**
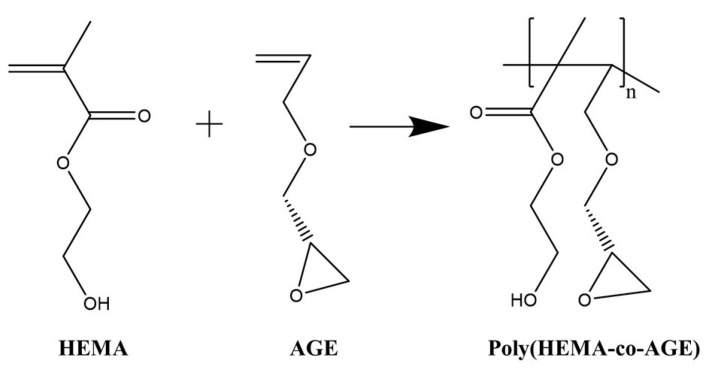
Mechanistic overview of HEMA and AGE monomer interactions during the formation of Poly(HEMA-co-AGE) cryogels.

Poly(HEMA-co-AGE) cryogels were synthesized via free radical cryopolymerization (Figure 6). Initially, 0.283 g of MBAAm was dissolved in 10 mL of distilled water. In a separate solution, varying volumes of HEMA (1900, 1750, or 1500 µL) and AGE (100, 250, or 500 µL) were mixed in 3.1 mL of distilled water. Both solutions were cooled in an ice bath before mixing.

After combining the two solutions, 20 mg of APS was added as the initiator, followed by 25 µL of TEMED to catalyze the polymerization reaction. The resulting precursor solution was poured between two glass plates and subjected to cryopolymerization at −12 °C for 24 h.

Following polymerization, the cryogels were thawed at room temperature and thoroughly washed with 200 mL of distilled water containing 0.02% sodium azide in 0.1 M phosphate buffer (pH 7.0) [32]. Finally, the cryogels were cut into membrane (disc) shapes and stored at 4 °C until further use.

### 4.3. Characterization Studies

This study employed a range of analytical techniques to systematically characterize Poly(HEMA-co-AGE) cryogels and compare them with conventional Poly(HEMA) cryogels. Swelling tests were performed to assess the water absorption capacity and network porosity, which are key parameters for evaluating the structural properties of the cryogels. The water retention capacity (WRC) was calculated using the following equation:

(1) $$\mathrm{WRC}\;(\%)\;=\;\frac{W_{s}-W_{d}}{W_{s}}\times 100$$

where:

*W_s_*: The weight of the swollen cryogel after equilibration in water.

*W_d_*: The weight of the cryogel after drying or centrifugation.

Fourier-transform infrared (FT-IR) spectroscopy was utilized to verify successful copolymerization and identify functional groups resulting from the incorporation of AGE into the polymeric network. FT-IR spectra were recorded using a Thermo Scientific Nicolet 6700 FT-IR (Waltham, MA, USA) spectrometer in the range of 4000–400 cm^−1^ with a resolution of 4 cm^−1^, using the attenuated total reflectance (ATR) technique. Scanning electron microscopy (SEM) and transmission electron microscopy (TEM) provided detailed observations of the cryogels’ surface morphology and pore architecture, offering valuable insights into their suitability for adsorption and enzyme immobilization applications. SEM images were obtained using a FEI/Quanta 450 FEG (Hillsborough, OR, USA) scanning electron microscope operating at 10–20 kV under high-vacuum mode. Cryogel samples were freeze-dried and sputter-coated with a thin layer of gold before imaging to enhance conductivity and resolution. In addition, TEM analysis was performed using a FEI Tecnai G^2^ Spirit BioTwin (Hillsborough, OR, USA) transmission electron microscope at an accelerating voltage of 200 kV. Cryogels were ultrathin-sectioned and placed on carbon-coated copper grids. Thermogravimetric analysis (TGA) was conducted to evaluate the thermal stability and decomposition behavior of the materials. TGA measurements (Shimadzu DTG-60H, Kyoto, Japan) were conducted using a platinum crucible under a nitrogen atmosphere with a heating rate of 10 °C·min^−1^ from 30 °C to 700 °C. Additionally, Brunauer–Emmett–Teller (BET) analysis was performed to determine the specific surface area and pore characteristics, critical parameters for assessing performance in surface-driven processes. BET surface area measurements were carried out using a Quantachrome Autosorb^®^ iQ-Chemisurface area and porosity analyzer (Boynton Beach, FL, USA). Before analysis, cryogel samples were degassed under vacuum at 90 °C for 12 h to remove moisture and volatile components.

### 4.4. Immobilization of Catalase

Catalase immobilization was performed using Poly(HEMA-co-AGE) cryogels synthesized with varying amounts of the functional monomer AGE (100, 250, and 500 μL). A catalase stock solution (10 mL, 1000 mg·L^−1^) was prepared, and its initial concentration was determined by measuring the absorbance at 280 nm using a UV–VIS spectrophotometer (TU-1810, Pgeneral, Beijing, China).

Cryogel discs were immersed in separate test tubes containing 10 mL of catalase solution for immobilization. At predetermined time intervals (2, 4, 8, 12, 16, and 24 h), one cryogel was removed from each tube, and the residual catalase concentration in the supernatant was again measured at 280 nm. A progressive decrease in absorbance indicated the successful adsorption of catalase onto the cryogel matrix.

This procedure was repeated systematically for all three cryogel formulations to assess the effect of AGE concentration on immobilization efficiency. Optimal immobilization times were determined based on the enzyme adsorption kinetics for each formulation. The time-resolved approach provided a quantitative understanding of enzyme uptake and allowed for the evaluation of immobilization performance as a function of monomer content. The immobilization capacity of catalase was calculated using the following equation and expressed in milligrams of enzyme immobilized per gram of cryogel (mg·g^−1^):

(2) $$\mathrm{Immobilization}\;\mathrm{Capacity}\;(q)\;=\;\frac{\left(C_{0}-C_{t}\right)\;\times \;V}{m}$$

where:

*C*_0_: The initial catalase concentration (mg·L^−1^),

*C_t_*: The catalase concentration in the solution at time *t* (mg·L^−1^),

*V*: The volume of the catalase solution (L),

*m*: The dry mass of the cryogel used (g).

This equation quantifies the amount of catalase removed from the solution and immobilized onto the cryogel matrix.

### 4.5. Determination of Catalase Activity for Free and Immobilized Forms

The catalytic activity of both free and immobilized catalase was evaluated by monitoring the decomposition of hydrogen peroxide (H_2_O_2_), a widely accepted substrate in catalase activity assays. The enzymatic reaction was tracked by measuring the characteristic decrease in absorbance at 240 nm using UV–VIS spectrophotometry.

A reaction medium containing 10 mM H_2_O_2_ in 50 mM phosphate buffer (pH 7.0) was prepared and maintained at 25 °C for the assay. Free and immobilized catalase samples were separately introduced into the reaction mixture. The reaction progress was monitored over a 1 min period by recording the decline in absorbance at 240 nm, assuming first-order reaction kinetics. The rate of absorbance change (ΔA_240_/min) served as an indicator of enzymatic activity, according to established protocols in the literature [34]. The detailed procedures and validation results for catalase activity determination using UV-VIS spectroscopy and permanganometric titration are provided in the Supplementary Materials.

### 4.6. Operational and Storage Stability of Immobilized Catalase

Operational stability is a crucial parameter for the industrial application of enzymes. To evaluate this, the catalytic activity of immobilized catalase was monitored over 15 consecutive reaction cycles at 25 °C. After each cycle, the Poly(HEMA-co-AGE) cryogel discs were regenerated by rinsing with 50 mM phosphate buffer (pH 7.0) to remove residual substrates and products. When not in use, the cryogels were stored at 4 °C. Additionally, the storage stability of both free and immobilized catalase was assessed over a seven-day period under identical buffer conditions [34].

### 4.7. Desorption and Reusability

To evaluate the reusability of Poly(HEMA-co-AGE) cryogels and assess enzyme desorption capacity, desorption experiments were conducted using a 1 M NaCl solution at pH 7.0. The cryogel disks were stirred continuously in a batch system for 1 h. After desorption, the cryogels underwent successive immobilization cycles to examine their operational stability. The desorption efficiency was determined using the equation below:

(3) $$DR\left(\%\right)=\frac{m_{catalase\;desorbed}}{m_{catalase\;adsorbed}}\times 100$$

## Figures and Tables

**Table 1:** Kinetic parameters for free and immobilized catalase.

	*K_m_* (mM)	*V_max_* (µmol·min^−1^)	*k_cat_* (min^−1^)	*k_cat_*/*K_m_* (µM^−1^·min^−1^)
Free enzyme	54.9	2433	60.83	0.0011
Immobilized enzyme	17.1	1108	51.30	0.0030

**Table 2:** Comparison of catalase immobilization on various cryogels.

Cryogel Type	IC (mg·g^−1^)	*K_m_* (mM) (Free/Imm)	*V_max_* (µmol min^−1^) (Free/Imm)	OS	SS	CR	Ref.
p(HEMA)-BPCGO	261.7 ± 11.2	9.9/11	357.1/769.2	100% (1st use), 34.4% (15th use)	65.12% (at 4 °C, 15 days)	100% (1st use), 90.1% (5th use)	[35]
Poly(HEMA-GMA)	298.7 ± 9.9	10.52/5.43	10,000/2500	100% (1st use); 31.0% (15th use)	70.0% (at 4 °C, 7 days)	100% (1st use), 87.21% (5th use)	[34]
p(HEMA-co-AGE)	49.3 ± 1.2	46/19	330/8.7	–	No significant decrease after 45 days	100% (1st use), 93.8 ± 1.2% (5th use)	[32]
Fe^3+^-poly(AAm-GMA)-IDA	12.99	–	–	–	–	100% (1st use), 96.7% (40th use)	[36]
Poly(HEMA-co-AGE)-250	356.3 ± 3.6	54.9/17.1	2433/1108	100% (1st use); 33.1% (15th use)	80.6% (at 4 °C, 35 days)	100% (1st use), 85.69% (5th use)	This study

IC: immobilization capacity, Imm: immobilized, OS: operational stability, SS: storage stability, CR: cryogel reusability, BPGDO: 4-biphenylchloroglyoxime, IDA: iminodiacetic acid.

## Data Availability

The original contributions presented in this study are included in the article. Further inquiries can be directed to the corresponding author.

## Supplementary Materials

The following supporting information can be downloaded at: https://www.mdpi.com/article/10.3390/gels11080634/s1, Figure S1: Comparative UV–VIS absorbance profiles of free and immobilized catalase at 240 nm, Figure S2: Correlation between UV–VIS and permanganometric methods for determining catalase activity, Table S1: Comparison of catalase activity values determined using UV–VIS spectroscopy and permanganometric titration for free and immobilized forms.
