# Supplementary material for: Cryogel-Immobilized Catalase as a Biocatalyst with Enhanced Stability Against Microplastics
Source: Gels. 2025 Aug 12;11(8):634. doi: 10.3390/gels11080634 (PMC12385229; doi:10.3390/gels11080634)
Supplement: Supplementary file 1 [file gels-11-00634-s001.zip › gels-3784751-supplementary.pdf]

Supplementary Materials

1. Determination of Catalase Activity via UV-VIS Spectroscopy

Catalase activity was measured spectrophotometrically by monitoring the decomposition of hydrogen peroxide (H_2O_2) at 240 nm. The assay was performed in 50 mM phosphate buffer (pH 7.0) at 25 °C.

Reagents and Conditions:

- Substrate: 10 mM H_2O_2 in 50 mM phosphate buffer
- Enzyme concentration: 0.05 $mg \cdot mL^{-1}$ (free and immobilized)
- Path length: 1 cm
- Total reaction volume: 3 mL
- Monitoring time: 1 min (with data points every 5 s)

Procedure:

1. The absorbance of the reaction mixture was recorded immediately after adding the enzyme.
2. The rate of H_2O_2 decomposition was calculated from the linear portion of the absorbance-time curve.
3. The molar extinction coefficient of H_2O_2 at 240 nm ($\varepsilon = 43.6\ M^{-1} \cdot cm^{-1}$) was used for activity calculations.

Equation Used:

Catalase activity (U) = $(\Delta A_{240}/\varepsilon \times t) \times V_{total} \times 10^6$

Where:

- ΔA_{420} is the change in absorbance per minute,
- $\varepsilon = 43.6\ M^{-1} \cdot cm^{-1}$,
- t is time in minutes,
- V_{total} is the reaction volume in L.

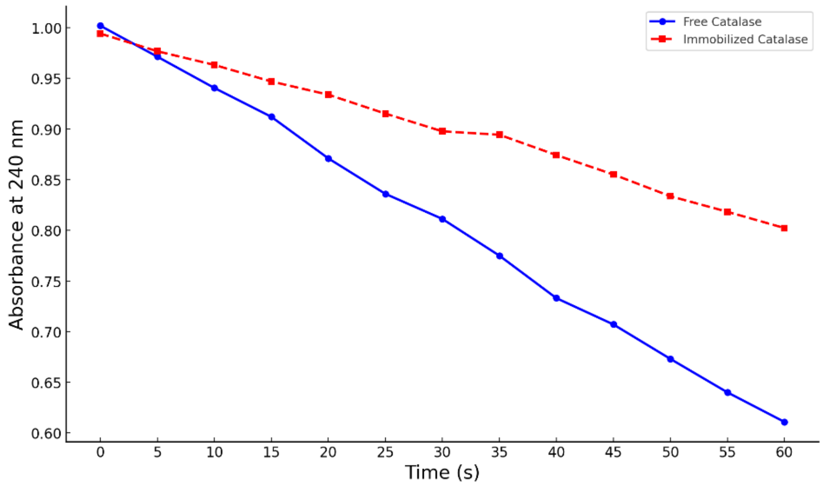

Figure S1. Comparative UV-VIS absorbance profiles of free and immobilized catalase at 240 nm.

2. Catalase Activity Determination via Permanganometric Titration (Validation Study)

To validate UV-VIS results, catalase activity was independently measured using the permanganometric method based on the volume of $KMnO_4$ required to titrate the residual H_2O_2.

Method Summary:

- After the enzymatic reaction (30 s), 2 mL of the reaction mixture was quenched with 2 mL of 2 M H_2SO_4.
- The remaining H_2O_2 was titrated with 0.01 M $KMnO_4$ until persistent pink color.

Reaction:

$$5H_2O_2 + 2KMnO_4 + 3H_2SO_4 \rightarrow 5O_2 + 2MnSO_4 + K_2SO_4 + 8H_2O$$

Equation for H_2O_2 concentration:

$$[H_2O_2] = (V_{KMnO4} \times N \times 17)/V_{sample}$$

Where:

- N = normality of $KMnO_4$,
- V_{KMnO_4} = volume of $KMnO_4$ used (mL),
- 17 = equivalent weight of H_2O_2.

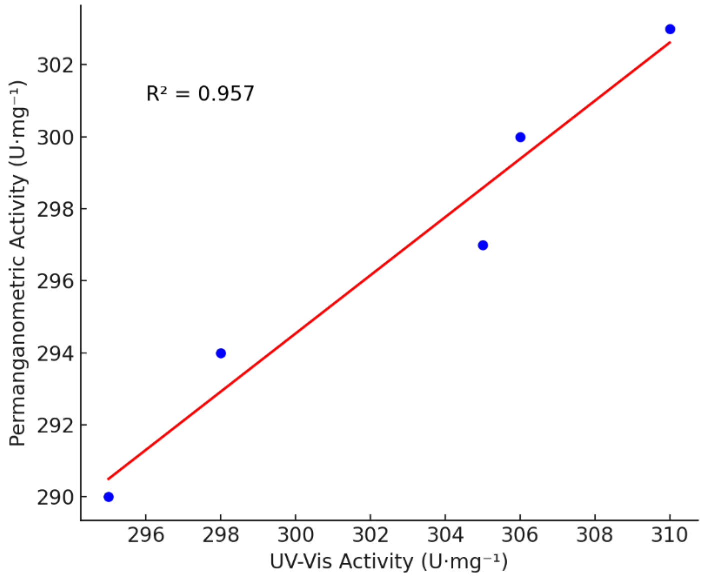

Figure S2. Correlation between UV-VIS and permanganometric methods for determining catalase activity.

Table S1. Comparison of catalase activity values determined by UV-VIS spectroscopy and permanganometric titration for free and immobilized forms.

Catalase Form	Activity (UV-VIS) ($U \cdot mg^{-1}$)	Activity (Permanganometric) ($U \cdot mg^{-1}$)	Deviation (%)
**Free Catalase**	305 ± 9.2	297 ± 11.4	2.62
**Immobilized Catalase**	211 ± 7.5	204 ± 9.0	3.32

The strong correlation between the UV-VIS spectrophotometric and permanganometric titration methods confirms the reliability of the primary method used in the manuscript. The UV-VIS method is suitable for determining the real-time and non-destructive activity of both free and immobilized catalase.
